# Roles of tumor-associated neutrophils in tumor metastasis and its clinical applications

**DOI:** 10.3389/fcell.2022.938289

**Published:** 2022-08-17

**Authors:** Man Yan, Minying Zheng, Rui Niu, Xiaohui Yang, Shifeng Tian, Linlin Fan, Yuwei Li, Shiwu Zhang

**Affiliations:** ^1^ Graduate School, Tianjin University of Traditional Chinese Medicine, Tianjin, China; ^2^ Department of Pathology, Tianjin Union Medical Center, Tianjin, China; ^3^ Nankai University School of Medicine, Nankai University, Tianjin, China; ^4^ Graduate School, Tianjin Medical University, Tianjin, China; ^5^ Department of Colorectal Surgery, Tianjin Union Medical Center, Tianjin, China

**Keywords:** metastasis, neutrophils, tumor-associated neutrophils, tumor microenvironment, prognostic potentials, antitumor immunity

## Abstract

Metastasis, a primary cause of death in patients with malignancies, is promoted by intrinsic changes in both tumor and non-malignant cells in the tumor microenvironment (TME). As major components of the TME, tumor-associated neutrophils (TANs) promote tumor progression and metastasis through communication with multiple growth factors, chemokines, inflammatory factors, and other immune cells, which together establish an immunosuppressive TME. In this review, we describe the potential mechanisms by which TANs participate in tumor metastasis based on recent experimental evidence. We have focused on drugs in chemotherapeutic regimens that target TANs, thereby providing a promising future for cancer immunotherapy.

## Introduction

In the last few decades, tremendous progress has been made in tumor immunology. Significant advances in immunotherapy, particularly in the use of checkpoint inhibitors, have been successful in targeting metastatic cancers. Although immuno-oncology treatments have gained considerable momentum, most metastatic cancers remain drug-resistant. This resistance is primarily due to innate immune cells such as neutrophils. Increasing evidence indicates that neutrophils increase resistance to clinical checkpoint blockade treatment, which is vital in establishing an immunosuppressive microenvironment and facilitating metastasis (Faget et al., 2021).

Metastasis, a primary characteristic of many malignancies, is associated with more than 90% of cancer-related deaths (Seyfried and Huysentruyt, 2013). Metastasis is the process by which tumor cells break away from their initial locations and are transported through the lymphatic or circulatory systems to form new tumors in other organs. The process involves several steps: 1) invasion of tumor cells into the neighboring parenchyma, 2) intravasation into the blood and/or lymphatic circulation, 3) survival within the circulatory or lymphatic systems, 4) extravasation into the distant parenchyma, and 5) subsequent survival and growth (Kitamura et al., 2015). Metastasis is associated with differentiation and tumor-associated neutrophils (TANs) that live in the tumor microenvironment (TME) (Lin et al., 2019). TANs constitute a major component of the TME and regulate tumor metastasis in all the five steps mentioned earlier.

TANs impair antitumor immunity and accelerate tumor growth and metastasis through the production of growth factors, chemokines, and inflammatory factors such as matrix metalloproteinase-9 (MMP-9), vascular endothelial growth factor (VEGF), high mobility group box 1 (HMGB1), and interleukin (IL)-17 (Bald et al., 2014; Minder et al., 2015; Li et al., 2019; Shaul and Fridlender, 2019). In recent years, the targeting of TANs has attracted increasing attention from researchers. This review provides an overview of the diversity and heterogeneity of TANs and describes the latest research progress on the potential mechanisms of TAN involvement in tumor metastasis. With a clearer understanding of the relationship between TANs and metastasis, treatment methods targeting TANs may provide a more promising approach to cancer intervention.

## TANs in tumors

Some studies have indicated that tumorigenesis follows a sequence of inflammatory events that resemble wound healing but without resolution (Hua and Bergers, 2019; Deyell et al., 2021). In response to early signs of inflammation, proinflammatory immune cells are recruited from the bloodstream and local resident cells. Evidence from mouse models and patients indicates that neutrophils are critical components of tumor-promoting inflammation in many tumor types (Galdiero et al., 2018; Shaul and Fridlender, 2019). Inflammation resolution and wound healing are hindered by genomic instability caused by neutrophils. For example, in breast cancer models, TANs produce reactive oxygen species that damage DNA and induce genetic instability, thereby promoting metastasis (Timaxian et al., 2021). Similarly, microparticles equipped with proinflammatory microRNAs (miR-23a and miR-155) are released by tissue-infiltrating neutrophils into intestinal epithelial cells. miRNAs enhance the formation of double-stranded breaks (DSBs) by causing the collapse of the replication fork. Their accumulation in the damaged epithelium hinders colonic repair and increases genomic instability (Butin-Israeli et al., 2019). A transgenic zebrafish tumor model indicated that TANs stimulate the early steps in tumor formation and progression. Direct live imaging revealed an innate inflammatory response at the pre-neoplastic stage. Zebrafish have been established as an indispensable *in vivo* model for cancer research as they can offer dynamic visualization of tumor growth *in vivo*. Their natural transparency combined with fluorescent labeling allows real-time observation of individual cells in a living model. For example, the T-cell leukemia model was the first zebrafish cancer model, allowing the direct monitoring of the initiation and expansion of leukemic cells using fluorescence microscopy (Langenau et al., 2003; Langenau et al., 2005). The rapid development of zebrafish genetic tools and imaging technologies has allowed researchers to better understand the processes that govern leukocyte activity during tumor initiation (Elliot et al., 2020).

**FIGURE 1:**
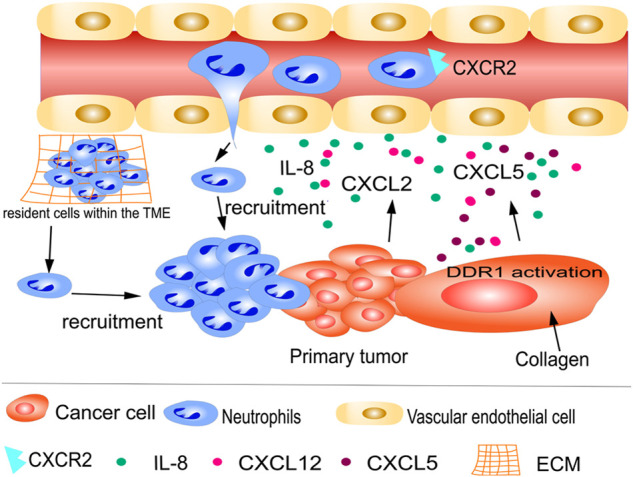
Recruitment of tumor-associated neutrophils (TANs) to primary tumors. Neutrophils from circulation and resident cells within the tumor microenvironment (TME) are recruited by cytokines, growth factors, and chemokines, such as IL-8 and CXCL2. Tyrosine kinase discoid domain receptor 1 (DDR1) is a key regulator of tumor cell metastasis. DDR1 activation by collagen in cancer cells is a significant stimulus for the synthesis of CXCL5, leading to the recruitment of TANs to primary tumors.

Similarly, as tumor cells build their environment to promote survival, they can trigger an inflammatory response, consequently recruiting more neutrophils into the TME (Wei et al., 2021). Neutrophils can infiltrate primary tumors; patients with higher numbers of infiltrating neutrophils have poorer prognoses and are more resistant to drug therapies (Arvanitakis et al., 2021; Kim et al., 2022; Tian et al., 2022). Neutrophil recruitment is triggered by immune mediators, including cytokines, growth factors, and chemokines (Fig. 1). Using immunohistochemistry, Schimek et al. demonstrated that neutrophils preferentially accumulated at locations of apoptotic tumor cells in 35 patients with colorectal cancer (CRC). In another CRC model, interleukin-8 (IL-8) was suggested to be secreted by apoptotic cancer cells, attracting neutrophils into the tumor where they interact with nearby macrophages, thereby establishing an immunologically unfavorable TME (Schimek et al., 2022). Additionally, in neutrophil-specific anterior gradient-2 (AGR2) knockout mice, a recent study reported that CRC cells aggressively attracted AGR2^+^ TANs, which enhanced CRC metastasis, through chemokine (C-X-C motif) ligand 2 (CXCL2) (Tian et al., 2022). Similarly, using co-cultures of neutrophils and tumors, polymorphonuclear myeloid-derived suppressor cells (PMN-MDSCs) were attracted to brain metastatic variants of breast cancer cells upon upregulation of CXCR2 and CXCL1 (Groth et al., 2021). Furthermore, tyrosine kinase discoid domain receptor 1 (DDR1) is a critical regulator, and its activation by collagen in tumor cells stimulates the synthesis of CXCL5, leading to the recruitment of TANs, invasion, and metastasis (Deng et al., 2021) (Figure 1).

A large body of data suggests that neutrophils recruited to tumor sites secrete cytokines, which induces adaptive immune response and leads to the recruitment of multiple immune cells, including B cells, T cells, and macrophages. Crosstalk between tumor and immune cells likely plays a vital role in maintaining an immunosuppressive TME (Chao et al., 2016; Shaul et al., 2021; Schimek et al., 2022). In another study, TANs with CD11b^+^/Ly6G^+^ cells were hypersegmented, more cytotoxic, and expressed higher levels of proinflammatory cytokines. They were attracted to tumor cells by transforming growth factor-beta (TGF-β) inhibitors through increased levels of neutrophil-attracting chemokines (Fridlender et al., 2009). Therefore, the function of TANs is influenced by the local microenvironment and other immune cells, demonstrating their diversity.

## Diversity and plasticity of TAN

Neutrophils have both pro- and anticancer functions. Transcriptomic analysis revealed that TANs have immunosuppressive properties that differ from those of healthy controls in humans and mice with cancer (Shaul and Fridlender, 2019). They can perform various functions related to their metabolic requirements, differentiation stage, and functional state (Shaul and Fridlender, 2019). A consensus has not been reached on the terminology for neutrophil heterogeneity and plasticity in cancer. However, researchers have attempted to distinguish between the subsets of neutrophils. Some molecules are hypothesized to be linked to the antitumor response, such as CD177 and CD101, or the protumor response, such as lectin-like oxidized low-density lipoprotein (LOX1), CD117, and programmed death-ligand 1 (PD-L1) (Noman et al., 2014; Condamine et al., 2016; Cheng et al., 2018; Evrard et al., 2018; Zhou et al., 2018; Zhu et al., 2018).

Neutrophils can also be distinguished based on their densities. Blood samples from patients with cancer contain two distinct populations: low-density neutrophils (LDNs) and high-density neutrophils (HDNs). LDNs exhibit pro-tumorigenic and immunosuppressive properties in cancer (Sagiv et al., 2015) and are composed of a mixture of mature and immature neutrophils. Conversely, HDNs are mature neutrophils with antitumor effects. In healthy controls, HDNs are predominant in circulating neutrophils (Sagiv et al., 2015).

Compared with macrophages, TANs are thought to be polarized into two distinct phenotypes: N1 antitumoral and N2 protumoral, as proposed by Fridlender et al*.* in 2009 (Fridlender et al., 2009). TGF-β released by the primary tumor can cause neutrophils to develop an N2-type pro-tumor phenotype, characterized by elevated arginase production and immunosuppressive effects on T cells (Fridlender et al., 2009; Eruslanov et al., 2014). Moreover, a recent study indicated that exosomal circPACRGL from CRC induces TGF-β1 production, resulting in neutrophil differentiation from N1 to N2 (Shang et al., 2020). Furthermore, some experiments have demonstrated that type I interferons (IFNs) can transform neutrophils into the N1 antitumor phenotype capable of cytotoxic activity against tumor cells that acquire antigen-presenting cell characteristics (Singhal et al., 2016; Siakaeva et al., 2019). Altering the TME may influence neutrophil plasticity and polarization. Triple-negative breast cancer models have indicated that Lin-28 homolog B (LIN28B) also enables neutrophil N2 conversion, maintaining the immunosuppressive environment by increasing the expression of programmed cell death one ligand 2 (PD-L2) (Qi et al., 2022). Similarly, in a CRC model, the cell migration-inducing protein KIAA1199 was produced by tumor recruitment of neutrophils with pro-tumor activity, indicating that KIAA1199 drives N2 pro-tumor polarization (Wang et al., 2022a). However, the association between N1/N2 neutrophils in mice and HDN and LDN classification in human patients is unclear because classification only considers functional conditions and not neutrophil maturation.

TANs with proven immunosuppressive functions are granulocytic myeloid-derived suppressor cells (G-MDSCs) or PMN-MDSCs, which act as neutrophils during multiple phases of maturation. Biochemical and gene expression profiling helped identify these cells as pathologically active immature myeloid cells that differ from normal myeloid cells (Bronte et al., 2016). The mechanism by which MDSCs differentiate from normal myeloid cells remains unknown, limiting our capacity to target them therapeutically in cancer. However, recent research employing single-cell RNA sequencing (scRNA-seq) has demonstrated that G-MDSCs appear in the spleen via an abnormal and unique neutrophil maturation pathway, contributing to their immunosuppressive features. However, the application of these findings to other tumors remains unexplored, as this study focused only on one type of cancer (Alshetaiwi et al., 2020). G-MDSCs are likely a mixture of immature and mature neutrophils with a low density. Their identification is generally confirmed by the presence of the following surface markers: CD15^+^, CD11b^+^, CD14^−^, HLA-DR^−^, and CD33; Furthermore, they are capable of inhibiting T cell activities and antitumor immunity (Cassetta et al., 2019). However, whether G-MDSCs should be considered a unique cell type or whether these results shed new light on neutrophil plasticity is debatable.

As shown in Table 1, most surface markers in LDNs, G-MDSCs, TANs immature neutrophil and mature neutrophil are similar. These “subgroups” display similar pro-tumor functions, rendering it challenging to distinguish between these “subgroups.” These neutrophil “subgroups” are likely a class of normal neutrophils stimulated by the tumor environment (McKenna et al., 2021). Identifying the cell subtypes within tumors is crucial to remove or repolarize their activity, enabling neutrophils to attack tumor cells rather than promote tumor metastasis.

**TABLE 1:** Main surface markers and function of LDNs, TANs, and G-MDSCs, immature neutrophil and mature neutrophil.

Neutrophil subtype	Function	Surface marker
Immature neutrophil	Pro-tumor	CD66^+^CD15^+^CD33^+^CD49d^−^CD101^+^CD10^−^CD16^+^ (Evrard et al., 2018; Mukaida et al., 2020)
Mature neutrophil	Anti-tumor	CD66^+^CD15^+^CD33^+^CD49d^−^CD101^+^CD10^+^CD16^+^ (Evrard et al., 2018; Mukaida et al., 2020)
LDN	Immunosuppressive activities	CD66b^+^ CD15^+^ CD33^+^ CD11b^+^ HLA-DR^+^ CD16^+^ (Hacbarth and Kajdacsy-Balla, 1986; Fu et al., 2014; Davis et al., 2017; Ui Mhaonaigh et al., 2019)
TAN (N2)	Promote the tumor progression and metastasis	CD66b^+^ CD15^+^ CD33^+^ CD11b^+^ CD16^+^ CD62L^+^ CD45^+^ (Fortunati et al., 2009; Cui et al., 2013; Eruslanov et al., 2014; Wu et al., 2014; Sionov et al., 2015; Uribe-Querol and Rosales, 2015; Shaul and Fridlender, 2019)
TAN(N1)	Suppress the tumor progression and metastasis	CD66b^+^ CD15^+^ CD33^+^ CD101+ CD10^+^ CD16^+^ (Yvan-Charvet and Ng, 2019; Mukaida et al., 2020)
G-MDSC	Suppress immune response and promote tumor progression in mouse model	CD66b^+^ CD15^+^ CD33^+^ CD11b^+^ CD14^−^ HLA-DR^−^ CD16^+^ (Khaled et al., 2014; Noy and Pollard, 2014; Ding et al., 2016; Cassetta et al., 2019; Shaul and Fridlender, 2019; Mukaida et al., 2020)

## Mechanisms underlying TAN-facilitated metastasis

### TANs promote tumor-cell invasion

Generally, highly invasive tumors lose their intrinsic polarity and become free from surrounding tissues (Savagner, 2010). Epithelial cells lose tight cell junctions and acquire mesenchymal characteristics by downregulating E-cadherin, a process known as the epithelial-mesenchymal transition (EMT). As a result, tumor cells that undergo EMT gain motility and aggressiveness as well as the capacity to rebuild the extracellular matrix (ECM) (Kalluri and Weinberg, 2009). Several studies have revealed that TANs may play a role in controlling EMT. As a natural inhibitor of MMP-9, the tissue inhibitor of metalloproteinase 1 (TIMP-1) exerts antitumor effects. However, recent studies have confirmed that neutrophil-secreted TIMP-1 promotes metastasis by inducing the EMT in breast cancer cells. Owing to the presence of the cluster of differentiation 90 protein (CD90) in these tumor cells, neutrophils produce more TIMPs, thereby promoting tumor development. Therefore, blocking CD90 or TIMP-1 significantly reduces the spread of cancer (Wang et al., 2019a). Similarly, Li et al. demonstrated that gastric cancer tissues exhibit high infiltration of TANs, especially at the invasive edge of the tumor, where IL-17 is expressed via Janus kinase 2 (JAK2)/signal transduction and activator of transcription 3 (STAT3) signaling, allowing them to increase migration, invasion, and EMT. Antibodies that neutralize IL-17a reduce tumor progression in gastric cancer (GC) cells (Li et al., 2019). Moreover, in an oral squamous cell carcinoma (OSCC) model, TANs also promoted EMT in OSCC cells by activating JAK2/STAT3 signaling (Hu et al., 2022).

**FIGURE 2:**
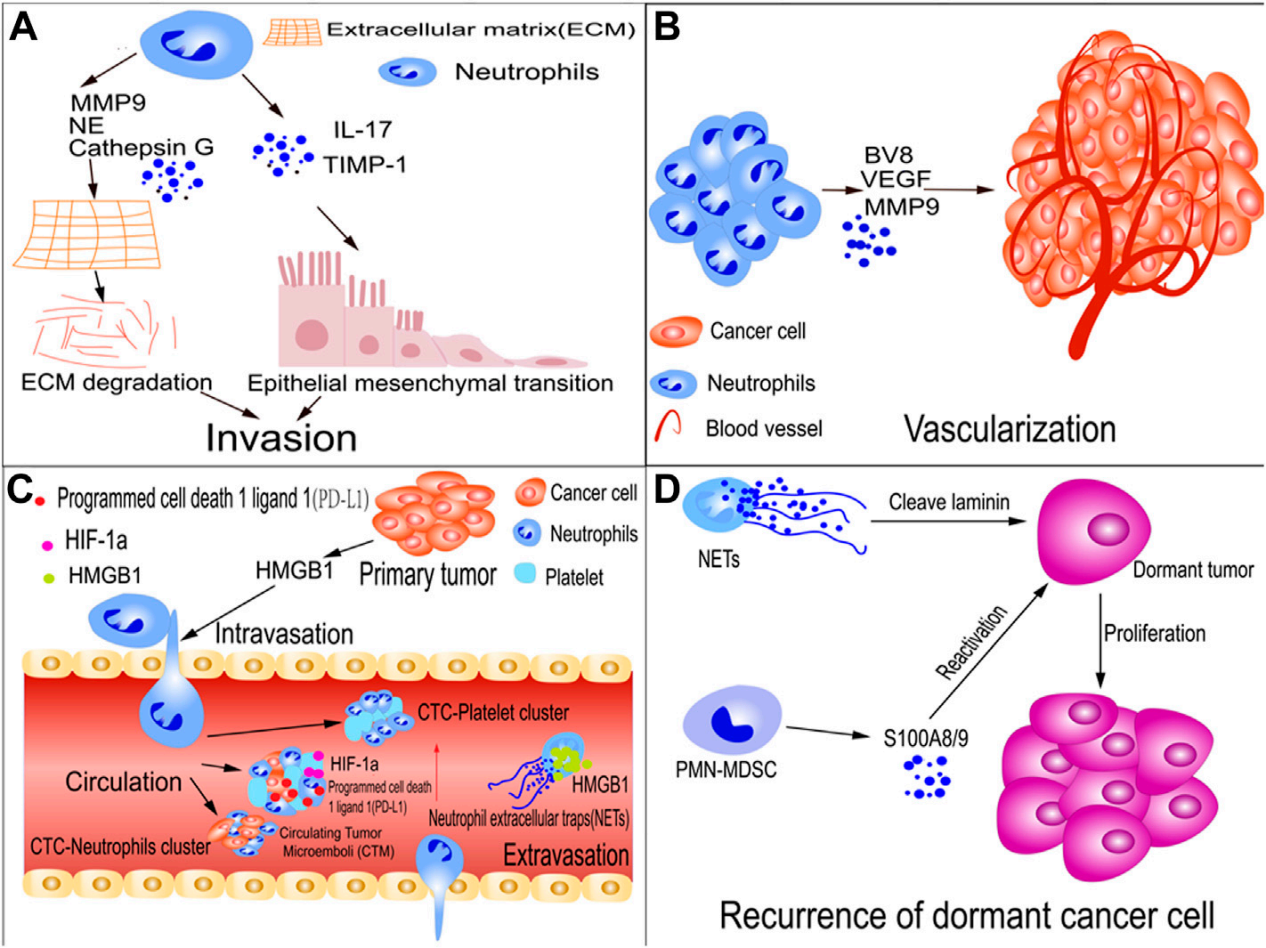
TAN roles in tumor metastasis. **(A)** TANs promote tumor-cell invasion; neutrophil-secreted TIMP-1 or IL-17 promote metastasis by inducing the epithelial-mesenchymal transition (EMT). Similarly, TANs cause cancer cells to undergo EMT by activating JAK2/STAT3 signaling. TANs secrete matrix metalloproteinase-9 (MMP-9), cathepsin G, and neutrophil elastase (NE), which degrade the extra-cellular matrix (ECM), thereby allowing tumors to invade adjacent tissues. **(B)** TANs promote tumor cell vascularization; neutrophils encourage tumor metastasis by releasing proteins such as MMP-9, vascular endothelial growth factor (VEGF), and BV8, which promote tumor angiogenesis. **(C)** TANs facilitate intravasation and extravasation of tumor cells and the survival of circulating tumor cells (CTCs); HMGB1 is released by tumor cells to recruit neutrophils, which assist tumor cells in invading blood vessels. CTC, CTC-neutrophil, and CTC-platelet clusters shield tumor cells from shear stress and natural killer (NK) cell assault. Hypoxia-inducible factor-1α (HIF-1α) expression is increased in circulating tumor microemboli (CTM) in the lungs. **(D)** Recurrence of dormant cancer cells; NETs cleave laminin, which results in the generation of an epitope. When a dormant cancer cell recognizes the epitope, signaling encourages tumor cells to awaken and proliferate. Polymorphonuclear myeloid-derived suppressor cells (PMN-MDSCs) awaken dormant tumor cells by S100A8/A9.

A recent study found that neutrophil extracellular traps (NETs), the DNA complexes generated by neutrophils to fight bacterial pathogens, may enhance GC growth, invasion, and migration. In patients with GC and postoperative abdominal infectious complications (AIC), NETs were reportedly released by neutrophils within the abdominal fluid (ascites) and omental tissues due to stimulation by postoperative AIC. NETs cause EMT in GC cells through the TGF-β signaling pathway, thereby exacerbating GC metastasis. This also explains the link between postoperative AIC and metastases after radical gastrectomy in patients with advanced GC. Additionally, using a transwell migration assay, AIC-induced NETs captured scattered GC cells to form NET-GC clusters, contributing to metastatic extravasation and implantation (Xia et al., 2022). However, another study showed that TANs inhibited EMT. In tumor-bearing mice, radiotherapy generates persistent DNA damage and subsequently triggers the secretion of the inflammatory chemokines CXCL1, CXCL2, and CCL5. These chemotactic factors recruit neutrophils to tumor sites and generate reactive oxygen species through phosphatidylinositol 3-kinase (PI3K)/protein kinase B (AKT)/zinc finger protein SNAI1 (Snail) signaling, inhibiting EMT and converting neutrophils to an antitumor phenotype (Liu et al., 2021a) (Figure 2A). Therefore, TANs appear to be involved in EMT regulation in cancer, and different types of neutrophils have different effects on EMT. In the future, the physiological polarization of TANs may significantly impact cancer immunotherapy.

Most solid tumors are encapsulated by an ECM composed of collagen, enzymes, and proteins that maintain tissue structure and function. However, they also serve as a hurdle for metastasis (Paolillo and Schinelli, 2019). Various proteases secreted by TANs, including cathepsins, MMPs, and neutrophil elastases (NE), are crucial for regulating ECM degradation. They can disrupt the barrier, allowing tumors to invade adjacent tissues (El Rayes et al., 2015; Ci et al., 2018; Wu et al., 2019) (Figure 2A).

### TANs promote tumor cell vascularization

When solid tumors reach a size threshold, various factors initiate the development of dense vasculature that provides a constant blood supply to the tumor. This process is called the angiogenic switch (Hanahan and Folkman, 1996). TANs serve as critical regulators of the angiogenic switch by eliciting the release of several proteases. Neutrophils are concentrated near the invasive edge of GC tissues and serve as a primary source of MMP-9, which promotes angiogenesis in GC cells (Li et al., 2017). MMP-9 promotes tumor angiogenesis by activating VEGF and VEGF receptor interactions in endothelial cells (Minder et al., 2015). Furthermore, prokineticin 2 (Bv8) triggers neutrophil-dependent angiogenesis, and inhibition of Bv8 activity limits angiogenesis and tumor development (Shojaei et al., 2007). A recent study showed that the transcription factor forkhead box protein O3a (FOXO3a) regulates the production of pro-angiogenic factors such as VEGF, MMP-9, and BV8 in neutrophils of IFN-deficient mice (Figure 2B). These pro-angiogenic factors play a crucial role in vascularization during tumor growth. Therefore, targeting FOXO3a holds promise as an anti-angiogenic therapy (Bordbari et al., 2021). Similarly, another study showed that nicotinamide phosphoribosyltransferase (NAMPT), a protein implicated in the downstream signaling of the granulocyte colony-stimulating factor receptor (G-CSF-R), is required for vascularization in a transplantable tumor model. Therefore, targeting neutrophils may be therapeutically possible (Pylaeva et al., 2019).

TANs help tumors disseminate distantly through the blood and lymphatic vessels. In the primary tumors of patients with OSCC, the presence of TAN-cancer cell compounds in areas of lymphangiogenesis indicates that TANs assist in tumor metastasis via lymphatic vessels (Lonardi et al., 2021).

### TANs promote tumor-cell intravasation

It is also crucial for cancer cells to gain access to the vasculature through the small holes in the vascular endothelium. In a mouse melanoma model, ultraviolet (UV) irradiation increased the aggregation of tumor cells along the surfaces of the blood vessels. HMGB1 is released by UV-damaged epidermal keratinocytes to recruit neutrophils, which assist tumor cells in invading the blood vessels (Bald et al., 2014) (Figure 2C).

### Neutrophils help circulating tumor cells survive in the peripheral circulation

Tumor cells must overcome multiple obstacles, including fluid shear stress, mechanical collisions, and immunosurveillance, to survive in the bloodstream (Lambert et al., 2017). Although several CTCs pass through the bloodstream, only a minimal fraction survive and spread to distant organs (Massagué and Obenauf, 2016). The tumor cells that survive in the bloodstream become more aggressive, and their ability to survive in the blood can be enhanced through various pathways. Numerous studies have demonstrated that the interplay between cancer cells and neutrophils increases tumor metastasis via the blood. Furthermore, neutrophils can communicate with CTCs to survive in the bloodstream. CTCs can form clusters of two to hundreds of cells to overcome the fluidic challenges of the bloodstream. These clusters have a 23–50-fold metastatic capacity compared to that of single CTCs in circulation. In patients with breast cancer, the abundance of CTC clusters was associated with adverse outcomes and metastasis (Aceto et al., 2014) (Figure 2C). CTCs are occasionally associated with leukocytes. Using single-cell RNA sequencing, Szczerba et al. analyzed a 4T1 mammary tumor model and human breast cancer. CTC-associated white blood cells were detected, most of which were CD11b^+^/Ly-6G^+^ neutrophils. Additionally, CTC neutrophil clusters have a greater tendency for metastatic spread than that of individual CTCs. Therefore, CTC–neutrophil clusters help increase survival and metastatic capacity (Szczerba et al., 2019) (Figure 2C). A recent study using an H22 tumor model reported that multipoint costriking nanodevices are required to kill primary tumors and prevent the dissemination of associated CTCs. Vascular cell adhesion molecule 1 (VCAM1) and glypican-3 are highly expressed on tumor cells and bind to neutrophils. The multipoint costriking nanodevice was developed using an anti-VCAM1 monoclonal antibody (mAb) and anti-Glypican-3 mAb, which can specifically identify and bind to their respective receptors overexpressed on the CTC membrane. Sorafenib and digitoxin located on the multipoint costriking nanodevice dissociated the CTC clusters, blocked the formation of CTC-neutrophil clusters, and destroyed the CTCs, thereby preventing migration (Mu et al., 2022).

Platelets may also protect tumor cells by forming platelet-rich thrombi around them. Platelets attract myeloid cells by secreting chemokines that shield tumor cells from shear stress and natural killer cells (NK), thereby allowing tumor cells to aggregate with platelets on the endothelium and accelerating tumor spread (Schlesinger, 2018). Platelets and neutrophils may also cooperate to shield CTCs from the vasculature via mechanical and immune-mediated destruction. Researchers discovered a pro-metastatic compound in several metastatic tumors consisting of CTC clusters, platelets, and neutrophils, known as circulating tumor microemboli (CTM).

Hypoxia is a vital factor in fostering CTM colonization in the lungs. Hypoxia-inducible factor-1α (HIF-1α) is highly expressed in the CTM, and its downregulation significantly alleviates hypoxia and ultimately inhibits metastasis (Du et al., 2022). Collectively, the presence of platelets and neutrophils in the bloodstream may increase the pro-metastatic activity of single CTCs. However, the mechanisms by which platelets and neutrophils contribute to CTC clusters mediating migration in different tumor types requires further investigation.

### TANs help tumor cells colonize and extravasate

In addition to spreading and surviving in blood vessels, CTCs adhere to the endothelium and cross the vascular system into surrounding tissues (Follain et al., 2020). Accumulating evidence indicates that neutrophils regulate tumor cell adhesion to endothelial cells and transmigrate to metastatic sites. Although they are critical for cancer metastasis, the mechanisms underlying extravasation remain poorly understood. In addition, cancer cells recruit TANs to the TME, where they release NETs. Hiroki et al. examined NETs around metastatic tumors using clinical specimens. Through the activation of EMT, high mobility group box protein 1 (HMGB1) derived from NETs enhanced tumor extravasation during metastasis in a murine model of liver metastasis (Kajioka et al., 2021) (Figure 2C).

### TANs and persistent growth, dormancy, and recurrence of tumor cells

Tumor cells become dormant upon transmigration to the surrounding tissues. Further, they stop proliferating or growing and evading immunosurveillance until the activation of a trigger mechanism (Vera-Ramirez et al., 2018; Shimizu et al., 2019). Dormant cancer cells are characterized by increased p38 mitogen-activated protein kinase (p38 MAPK) and decreased extracellular signal-regulated kinase 1/2 (ERK1/2) activity; the p38 MAPK^high^/ERK^low^ phenotype is commonly used as a marker of dormancy (Recasens and Munoz, 2019). In addition, according to a recent study, some researchers have synthesized the hallmarks of dormant cancer cell states, including niche dependence, cell cycle arrest, drug resistance, immune evasion, metastatic relapse, and reversibility. Dormant cancer cells are partly influenced by the local environment or niche in which they are located (Phan and Croucher, 2020). Overall, targeting dormant tumor cells is contingent on the tumor and its resident TME.

Despite being an essential member of the TME, the involvement of neutrophils in tumor dormancy remains unclear. Studies on animal models have revealed that neutrophils contribute to tumor awakening and recurrence. For example, in a dormant breast cancer metastasis model, sustained smoking or lung infections triggered NET formation at the dormancy site in cancer cells. Neutrophils expelled the extracellular DNA trap net, covered with proteases such as NE and MMP-9s. NETs initiated matrix remodeling through cleavage of laminin, resulting in the generation of an epitope. The recognition of the epitope by the dormant cancer cells activates the signaling pathways that drive the proliferation of tumor cells (Albrengues et al., 2018). Michela et al. demonstrated that PMN-MDSCs awaken dormant tumor cells through stress- and inflammation-dependent mechanisms. Stress hormones cause massive secretion of proinflammatory S100A8/A9 compounds from PMN-MDSCs via β2-adrenergic receptors. Accumulation of oxidized lipids in neutrophils is caused by these proteins, which in turn stimulate an increase in fibroblast growth factors and induce the proliferation of dormant tumor cells (Perego et al., 2020) (Figure 2D). Overall, further research is required to elucidate the mechanisms controlling dormancy in human cancer metastasis.

### TANs prepare pre-metastatic niches for tumor cells

Primary tumors can establish favorable locations for metastasis to distant organs, called the pre-metastatic niche (PMN) (Kaplan et al., 2005). Studies have clarified that neutrophils play a crucial role in establishing PMNs. At these sites, neutrophils are recruited by multiple tumor-secreted factors, thereby establishing an immunosuppressive environment that aids the survival and metastasis of tumor cells. The presence of these neutrophils creates a route for recruiting tumor cells from the bloodstream and tissues into the PMN (Seubert et al., 2015; Wculek and Malanchi, 2015; Lee et al., 2019).

Primary tumors secrete multiple cytokines and chemokines to induce PMNs. For example, using a model PMN involving a hyaluronic acid (HA)-based gel loaded with CXCL12 (CLG), the tumors were revealed to induce PMNs in target organs, producing the tumor-derived factor CXCL12, which induces the recruitment of neutrophils and attracts CXCR4^+^ tumor cells both *in vivo* and *in vitro* (Ieranò et al., 2019). Furthermore, in a mouse metastatic CRC model, KIAA1199 facilitated the infiltration of immunosuppressive neutrophils into the liver. Mechanistically, cell migration-inducing hyaluronidase 1 (KIAA1199) activated the TGFβ signaling pathway by interacting with TGFβ receptor ½ (TGFBR1/2) to stimulate CXCL1 and CXCL3 production, thereby driving neutrophil aggregation in the liver and resulting in the suppression of the antitumor immunity of CD8^+^ T cells. Therefore, blocking KIAA1199 may hinder the development of tumor metastasis (Wang et al., 2022a).

**FIGURE 3:**
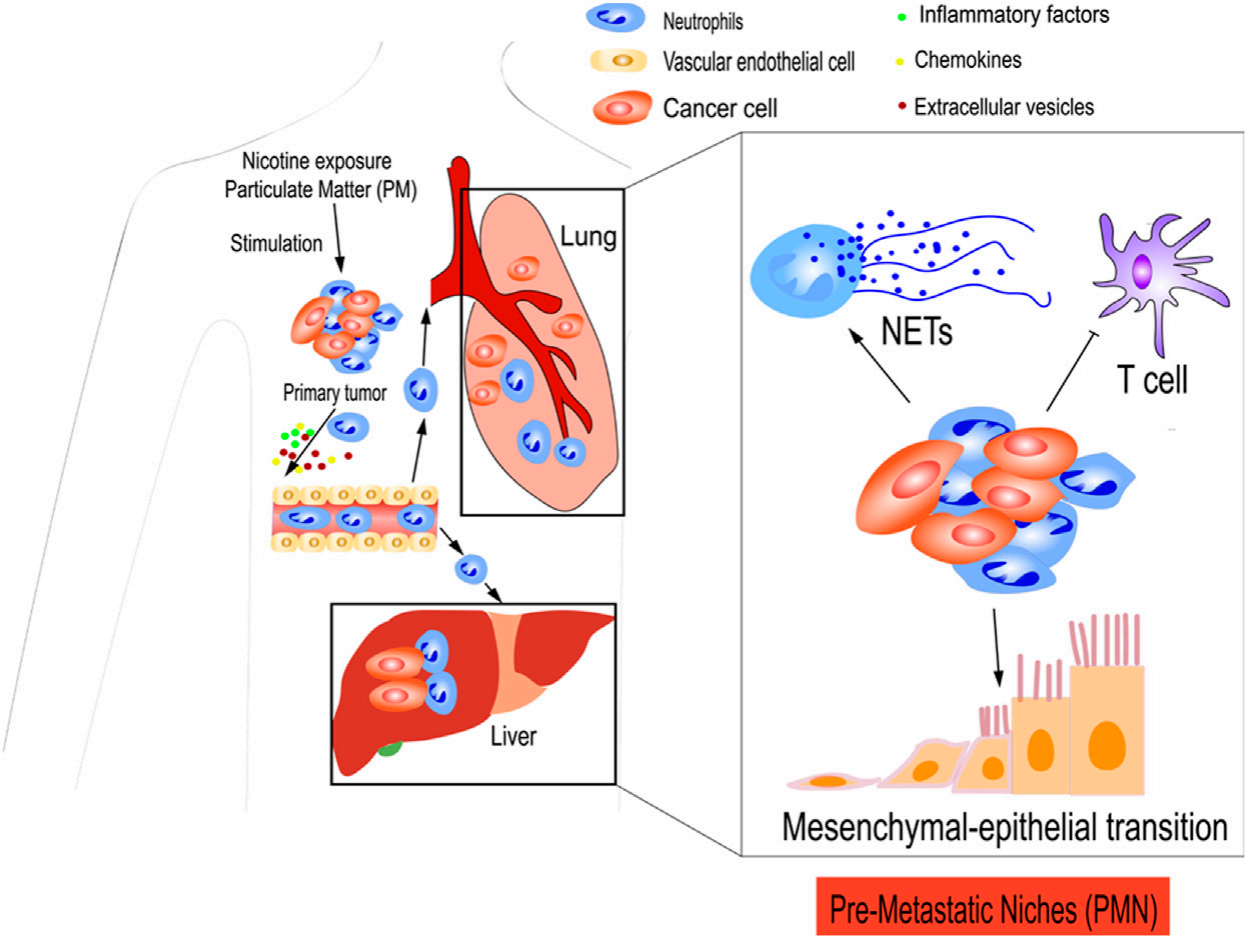
TANs prepare pre-metastatic niches (PMNs) for tumor cells. Primary tumors secreted various cytokines, chemokines, and inflammatory factors to establish PMN. CXCL12, CXCL1, and CXCL3 from tumors cause the aggregation of immunosuppressive neutrophils in the PMN, which result in the suppression of antitumor immunity of T cells. Neutrophils recruited to the PMN may release NETs that facilitate the trapping of cancer cells. Extracellular vesicles (EVs) have an important role in the formation of the PMN, which facilitates metastasis. Furthermore, nicotine exposure and particulate matter (PM) can also induce neutrophil recruitment and PMN formation. Nicotine exposure promotes the MET, thereby restoring some epithelial features and contributing to early colonization of the PMN.

In addition to cytokines and chemokines, inflammatory factors play a role in establishing PMNs. Lung mesenchymal stromal cells (LMSCs) are triggered by Th2-type cytokines to produce large amounts of complement component 3 (C3) proteins, which increase neutrophil recruitment of highly expressed C3a receptors to the PMN, ultimately promoting metastasis. As the number of NETs increases, more circulating tumor cells may be captured in the lungs and colonize the area. Targeting the Th2-type cytokine–STAT6–C3–NETs axis may offer a promising method for preventing lung metastasis in breast cancer (Zheng et al., 2021). In addition, NETs have been identified in the omenta of mice with ovarian tumors before metastasis and in the omenta of women with early-stage ovarian tumors. In orthotopic ovarian cancer models, tumor-derived factors attract neutrophils to the pre-metastatic omentum, generating NETs and facilitating the capture of ovarian cancer cells (Lee et al., 2019) (Figure 3).

Extracellular vehicles (EVs) are released by tumor cells into the bloodstream. They carry specific molecules, such as DNA and RNA, which contribute to the signaling between cells in the TME. Exosomes are a subset of extracellular vesicles (EVs). Recent studies have shown that tumor cells release exosomes that play a role in PMN formation, thereby promoting metastasis. For example, Meiyan et al. reported that LIN28B secreted by tumor cells attracted neutrophils to the lung and established an immunosuppressive PMN in a triple-negative breast cancer model and patients with breast cancer. LIN28B induced IL-6 and IL-10-mediated neutrophil N2 conversion in the lung PMN, which increased the expression of T cell exhaustion ligands and inhibited the activity of T cells. This promotes the spread of breast cancer to the lungs. Furthermore, they demonstrated that breast cancer-released exosomes with low let-7s are critical for neutrophil recruitment and LIN28B-induced lung PMN formation (Qi et al., 2022). In addition, Maximiliane et al. reported that through genetic ablation of the BAG6 protein and disruption of p53 acetylation, melanoma cells secrete EVs, which form a PMN in distant tissues that recruit N2 neutrophils and tumors, promoting metastasis and progression (Schuldner et al., 2019). Therefore, EVs play a critical role in the process of PMN, which strengthens the connection between primary tumor cells and distant organs, facilitating the metastasis of tumor cells to secondary organs. A deeper understanding of the mechanism by which tumor-derived exosomes regulate PMN is essential for future research.

In addition, TANs can be used to prepare tumor cell sites using other methods. For example, chronic nicotine exposure plays a critical role in the development of pulmonary PMN. N2-neutrophils stimulate the secretion of the STAT3-activated glycoprotein lipocalin-2 (LCN2) in the PMN, which encourages cancer cells to undergo MET, restore epithelial features, and contribute to early colonization in the PMN (Tyagi et al., 2021). Another study demonstrated that TNF receptor-associated factor 6 (TRAF6) accumulates via autophagic degradation of tripartite motif-containing protein 37 (TRIM37) in lung epithelial cells as a result of particulate matter (PM), which is essential for initiating neutrophil recruitment and PMN formation in the lungs (Liu et al., 2021b) (Figure 3).

Overall, primary tumors can establish PMN for metastatic seeding. However, the cellular mechanisms influenced by TME are complicated. A better understanding of the molecular mechanisms underlying PMN is essential for the development of specific therapeutic strategies targeting TANs.

## Prognostic value of TANs

Multiple studies have evaluated the prognostic value of the neutrophil-to-lymphocyte ratio (NLR) in a variety of cancers, including breast cancer (Krenn-Pilko et al., 2014), CRC (Grenader et al., 2016), non-small cell lung cancer (NSCLC) (Wang et al., 2019b), hepatocellular carcinoma (HCC) (Terashima et al., 2015), and melanoma (Cohen et al., 2020). Most studies have correlated a high NLR with poor prognosis. For example, a systematic meta-analysis of 100 studies exploring the prognostic value of NLR in patients with solid tumors emphasized that a high NLR was negatively associated with overall survival (OS) (Templeton et al., 2014). Low NLR is associated with improved survival rates, which was confirmed in 187 metastatic melanoma patients treated with ipilimumab (Ferrucci et al., 2015). Furthermore, in advanced NSCLC patients receiving nivolumab, the NLR has been proven to be a useful predictor of disease progression at 2 and 4 weeks after treatment (Nakaya et al., 2018). Ryoichi et al. indicated that the NLR is a valuable marker for predicting short-term outcomes in gastric cancer patients (Miyamoto et al., 2018). Neutrophils account for a considerable proportion of immune infiltration in many cancer types; thus, several studies have examined the prognostic value of quantifying tumor-infiltrating neutrophils.

The presence of TANs is associated with poor prognoses in several studies of advanced GC (Kim et al., 2022), early-stage melanoma (Jensen et al., 2012), head and neck cancer (Trellakis et al., 2011), and HCC (Li et al., 2011). However, the prognostic relevance of TANs in some types of cancer, such as CRC, remains controversial. Shaobo et al. reported that TANs promoted the metastasis of CRC cells through an AGR2-CD98hc-xCT-mediated pathway and that patients with CRC with an increased abundance of AGR2^+^ TANs had a poorer prognosis (Tian et al., 2022). In contrast, other studies have indicated that myeloperoxidase (MPO)^+^ neutrophil infiltration is associated with a favorable prognosis in CRC (Berry et al., 2017; Wikberg et al., 2017). Therefore, further research on neutrophil infiltration and its prognostic significance is required. Neutrophils play a vital role in the effectiveness of immune checkpoint inhibitors (ICIs). In one case, neutrophils in NSCLC suppressed T-cell immune function, leading to the failure of ICI therapy and their corresponding efficacy. (Kargl et al., 2019). However, the potential for neutrophil resistance to clinical immune checkpoint blockade treatments has recently been observed. ICIs used in combination with TANs reduce the effectiveness of anti-angiogenic drugs (Faget et al., 2021). Therefore, further exploration of the clinical implications of the role of neutrophils in ICI response may allow researchers to gain a deeper understanding of TAN biology and its effects on patients. Conflicting reports exist on the effects of neutrophils on chemotherapy and radiotherapy. 5-Fluorouracil (5-FU) and gemcitabine eliminate immunosuppressive neutrophils and activate neutrophil inflammasomes and IL-1β secretion, promoting chemotherapy resistance (Bruchard et al., 2013). In contrast, the neutrophil count can also indicate a good prognosis for patients with high-grade ovarian cancer (Posabella et al., 2020). Similar to chemotherapy, neutrophils have paradoxical effects on radiotherapy (Schernberg et al., 2017; Liu et al., 2021a). Therefore, whether the effect of neutrophils in response to chemotherapy and radiotherapy is beneficial or harmful remains controversial and may depend on the type, stage, or nature of cancer treatment.

## Strategies for targeting neutrophils

The results of these studies strongly suggest that neutrophils play a significant role in each step of the metastasis cascade. Several preclinical and clinical studies have shown that therapeutic targeting prolongs survival and decreases the risk of metastasis. Therefore, TANs may serve as potential therapeutic targets.

Next, we discuss current agents in terms of various mechanisms, such as inhibition of TAN survival, reprogramming of pre-tumor neutrophils into an antitumoral phenotype, and inhibition of TAN recruitment. The relevant drugs are listed in Table 2.

**TABLE 2:** Clinical trials of agents targeting TANs.

Compound	Target	Combination partner	Tumor type	Phase	Ref. or trial no.
Agents that inhibit TAN survival					
DS-8273a	PMN-MDSCs	Monotherapy	Head and neck tumor	3	Dominguez et al. (2017)
Gemcitabine	PMN-MDSCs	Monotherapy	NSCLC	3	NCT03302247
Fluorouracil (5-FU)	MDSC	Bevacizumab Anakinra	CRC	2	Isambert et al. (2018)
Agents that inhibit TAN recruitment					
SX-682	CXCR2	Pembrolizumab	Melanoma	1	NCT03161431
Reparixin	CXCR1	Paclitaxel	Triple-negative breast cancer	2	NCT02370238
Agents that reprogram pre-tumor neutrophils into an antitumoral phenotype					
Fresolimumab	TGF-β	Monotherapy	Renal cell carcinoma	1	Lacouture et al. (2015)
	TGF-β	Radiation therapy	Metastatic breast cancer	Completed	(Formenti et al., 2018)
	TGF-β	Monotherapy	High-grade gliomas	2	den Hollander et al. (2015)
Galunisertib	TGF-β	Galunisertib	HCC	2	Kelley et al. (2019)
		Sorafenib			
	TGF-β	Durvalumab	Metastatic pancreatic cancer	1	Melisi et al. (2021)

### Agents against TAN survival

DS-8273a, an agonistic death receptor 5 (TRAIL-R2) antibody, can induce apoptosis in various tumor cells while sparing vital normal cells. Two TRAIL receptors, TRAIL-R1 (DR4) and TRAIL-R2 (DR5), induce apoptosis (de Miguel et al., 2016). Researchers have used DS-8273a to selectively target MDSCs in a phase I study of patients with stage III head and neck squamous cell carcinoma. In patients with increased numbers of circulating PMN-MDSCs (CD11b+/CD14+/CD33+/CD15+; low-density fraction cells), treatment with DS8273a decreased their numbers (Dominguez et al., 2017). However, TRAIL receptor agonists are subject to further investigation in terms of their safety and tolerability. Gemcitabine, a nucleoside analog, is an effective antitumor drug. During DNA replication, its incorporation into DNA triggers strand termination leading to cell death. In patients with pancreatic cancer, PMN-MDSCs (CD11b^+^/CD14^-^/CD33^+^/HLA^−^/DR^−^) were significantly reduced after gemcitabine treatment. However, following the resting phase, the effect of the treatment was attenuated after terminating the administration of gemcitabine. This suggests that continuous administration of gemcitabine is required to achieve a lasting effect on PMN-MDSCs (Eriksson et al., 2016). Similarly, in a phase II clinical trial, gemcitabine increased the efficacy of nivolumab by killing MDSCs to reduce immunosuppression in stage IIIB NSCLC (NCT03302247). Capecitabine, a 5-FU prodrug, selectively induces MDSC death *in vitro* and *in vivo* more strongly than gemcitabine. MDSCs were eliminated by 5-FU, increasing the secretion of IFN-γ by tumor-specific CD8^+^ T cells that infiltrated the tumor and induced an antitumor response *in vivo* (Vincent et al., 2010). In a phase II clinical trial of patients with stage IV CRC, 5-FU plus bevacizumab and anakinra resulted in an approximate doubling of the median progression-free survival and a manageable safety profile (Isambert et al., 2018). However, DS-8273a, capecitabine, and gemcitabine have unavoidable side effects such as unselective neutrophil depletion. Therefore, developing agents that preferentially target N2 neutrophils is essential to minimize toxicity. However, further research is required before substantial clinical applications can be explored.

### Agents inhibiting neutrophil recruitment

As mentioned earlier, TME tumors secrete chemokines that attract tumor-associated neutrophils to the tumor sites. Therefore, a TAN-targeting anticancer therapeutic approach could include disrupting signals that attract neutrophils. The CXCR2 signaling pathway plays a critical role in mediating the movement of neutrophils from the bone marrow into the bloodstream and subsequently into the peripheral tissues. Numerous studies have revealed the efficacy of CXCR2-targeted therapy, which has initiated early-phase clinical trials. This therapy works through the use of SX-682, a small-molecule inhibitor of CXCR1 and CXCR2, which retards tumor development. In tumor-bearing mice, SX-682 reduced the infiltration of MDSCs into tumors and increased activated CD8^+^ T cells, thereby inhibiting tumor growth (Yang et al., 2021). Moreover, in head and neck cancer models, SX-682 decreased tumor infiltration caused by PMN-MDSCs and increased tumor infiltration and activation of adoptively transferred murine NK cells (Greene et al., 2020). However, further research on SX-682 in clinical trials is required. In stages III and IV melanoma patients, SX-682 was evaluated to determine whether it could block cancer cells from attracting MDSCs (NCT03161431). Identifying and elucidating the mechanism by which blocking CXCR2 may affect tumor growth is of particular interest. Reparixin is a non-competitive allosteric CXCR1/CXCR2 inhibitor. In a mouse cisplatin-induced acute kidney injury (AKI) model, it protected the kidneys by decreasing inflammatory cytokines and neutrophil infiltration (Liu et al., 2020). However, the applications of reparixin have not been extensively studied in the clinical treatment context. Reparixin is currently being evaluated in combination with paclitaxel chemotherapy versus paclitaxel alone in patients with metastatic triple-negative breast cancer for further evaluation of their progression-free survival.

### Reprogramming pre-tumor neutrophils into an antitumoral phenotype

Given the critical involvement of neutrophils in the immune system, whether the systemic elimination of neutrophils results in deleterious consequences during long-term treatment remains unknown. Although not studied extensively, pre-tumor neutrophils can reportedly be reprogrammed into antitumor neutrophils. The mechanism underlying this is through TGF-β-blocking antibodies, which enhance the tumor-killing activity of neutrophils. For example, mesoporous silica nanoparticles loaded with SB525334, an inhibitor of the TGF-β1 receptor, were designed in mouse models of pancreatic cancer. Local inhibition of TGF-β increases neutrophil polarization towards an anticancer phenotype in the TME and induces long-term antitumor memory (Peng et al., 2022). Similarly, another study revealed that a novel CRC-derived exosomal circPACRGL facilitated TGF-β1 expression and induced differentiation of N1 to N2 in mouse models (Shang et al., 2020). Moreover, in H22 mice, a novel nanovaccine reportedly changed the protumoral N2 phenotype of neutrophils to the antitumor N1 phenotype in the TME. The effect achieved complete tumor regression (83%) and prolonged survival (Wang et al., 2022b). However, further randomized clinical studies are warranted to validate the potential use of TGF-β in clinical practice. Additionally, fresolimumab was well tolerated and used in participants with osteogenesis imperfecta (OI) type IV, as well as a TGF-β-neutralizing antibody (Song et al., 2022). In phase I/II trials, fresolimumab was shown to have an acceptable safety profile and favorable systemic immune response in patients with melanoma, renal cell carcinoma (Lacouture et al., 2015), and metastatic breast cancer (Formenti et al., 2018). Zr-fresolimumab penetrated recurrent high-grade gliomas well but did not provide any clinical benefits (den Hollander et al., 2015). Clinical trials of fresolimumab are more commonly associated with TGF-β neutralization. Detailed studies are lacking on the functional reprogramming of neutrophils, which may have significant benefits for future tumor therapy.

Several clinical trials have demonstrated the tolerability and activity of single-agent or combination chemotherapy. Galunisertib is a small-molecule selective inhibitor of TGFβ receptor I. Recently, in a phase II trial with HCC, the combination of galunisertib and sorafenib showed acceptable safety and a longer OS outcome (Kelley et al., 2019). However, galunisertib plus durvalumab provided a disease control rate of 25% in patients with metastatic pancreatic cancer. Median OS and progression-free survival were 5.72 months (95% CI: 4.01–8.38) and 1.87 months (95% CI: 1.58–3.09), respectively. Biomarkers did not correlate with treatment outcomes, and this analysis was limited by the relatively small sample size (Melisi et al., 2021). Expanding the number of clinical samples is required to further evaluate their clinical value.

## Conclusion and perspectives

Cancer has a high incidence and mortality rate worldwide and is one of the most significant public health problems. While metastasis is a marker for malignancy, the results achieved with existing treatments are unsatisfactory because most aim to eliminate the tumor cells. However, successful seeding of metastases is greatly dependent on nonmalignant cells within the TME (Güç and Pollard, 2021). As the major components of the TME, TANs promote tumor progression and metastasis through multiple mechanisms. Therapeutic strategies targeting TANs may offer potential therapeutic opportunities contingent on improving our understanding of the relationship between TANs and metastasis. Targeting the pro-metastatic components of the TME to re-establish a healthy environment will undoubtedly offer viable avenues for tumor treatment.

Although these treatments are effective, several fundamental hurdles remain to be overcome. First, TANs exhibit remarkable heterogeneity and plasticity. Although they are usually considered the N2 type, they can display both tumoricidal and protumoral functions. The mechanism by which these phenotypes switch during tumor progression is not entirely understood. There are diverse subsets of neutrophils, including immature, mature, low-density, and high-density. Surface markers on neutrophils constantly change based on the influence of the TME; therefore, they can exhibit pro- or antitumor effects (Hsu et al., 2019). In particular, neutrophil metabolism is regulated by microenvironmental exposures that play different or contrasting roles, varying from killing tumor cells to supporting them. Although the metabolism of tumor cells has been extensively studied, the effect of cancer on neutrophil metabolism remains insufficiently understood. Further experiments are required to determine the mechanism by which TAN metabolism should be targeted to enhance cancer immunotherapy.

Second, the TME is a complex system consisting of several cellular and non-cellular components. Neutrophils, one of the main regulators in the TME, do not function alone but interact with other components. During metastasis, they are intricately linked to the surrounding matrix and to each other (Mao et al., 2021). A recent study reported that IL-8, released by apoptotic tumor cells, recruits neutrophils to the tumor, where they induce macrophages to exhibit an M2-like phenotype in both primary patient-derived and established CRC cells. Crosstalk between TANs and adjacent macrophages creates an immunosuppressive TME (Schimek et al., 2022). Overall, the roles of the components of the TME and the complex interactions between them remain largely unknown but are promising prospects for immunotherapy.

Third, the prediction and detection of micrometastases are major clinical problems. Cancer cells can escape from primary sites and spread to other organs, where they can remain dormant and clinically undetected for a long time. The triggers that cause these largely dormant cells to reawaken, proliferate, and spread are not fully understood. Micrometastases may appear months, years, or even decades before diagnosis in certain cancers (Hu et al., 2020). Understanding the mechanism by which tumors enter and exit dormancy is crucial to providing patients with effective treatment. Neutrophils can activate dormant cells and promote tumor metastasis. For example, in mouse models, a recent study showed that sustained inflammation induces the formation of NETs, which awakens dormant cancer cells by releasing NE and MMP-9. These neutrophil-associated enzymes sequentially cleave laminin, which induces the proliferation of dormant cancer cells by activating integrin α3β1 signaling (Albrengues et al., 2018). However, in most cases, dormant tumor cells can only be investigated through animal studies. Furthermore, detecting individual dormant tumor cells using current techniques is challenging. Further evidence is required to examine the process by which neutrophils awaken dormant tumor cells and disseminate them to secondary organs. A better understanding of this process may pave the way for the clinical exploitation of neutrophils.

In conclusion, this study provides a detailed review of the interactions between TANs and tumor cells during tumor metastasis. TANs are a potential target that could change the future landscape of cancer treatment; however, numerous hurdles remain unexplored. We believe that our review will encourage future research on the development of TAN-targeting drugs for cancer treatment.

## Glossary

AIC: abdominal infectious complication
AGR: neutrophil-specific anterior gradient
CRC: colorectal cancer
CTCs: circulating tumor cells
CTM: circulating tumor microemboli
DDR1: discoid domain receptor 1
DSBs: double-strand breaks
ECM: extracellular matrix
EGs: empty gels
EMT: epithelial-mesenchymal transition
EVs: extracellular vesicles
FOXO3a: forkhead box protein Oa
GC: gastric cancer
G-MDSCs: granulocytic myeloid-derived suppressor cells
HA: hyaluronic acid
HCC: hepatocellular carcinoma
HDNs: high-density neutrophils
HIF-1α: hypoxia-inducible factor-1α
HMGB1: high mobility group box 1
ICIs: immune checkpoint inhibitors
IFN: interferon
LDNs: low-density neutrophils
LMSCs: lung mesenchymal stromal cells
MDSCs: myeloid-derived suppressor cells
MET: mesenchymal-to-epithelial transition
MMP: matrix metalloproteinases
NAMPT: nicotinamide phosphoribosyltransferase
NE: neutrophils elastases
NETs: neutrophil extracellular traps
NSCLC: non-small cell lung cancer
OSCC: oral squamous cell carcinoma
PMN-MDSCs: polymorphonuclear myeloid-derived suppressive cells
PMNs: pre-metastatic niche
PM: particulate matter
TANs: tumor-associated neutrophils
TGF-β: transforming growth factor beta
TIMP-1: tissue inhibitor of metalloproteinases 1
TME: tumor microenvironment
UV: ultraviolet
VCAM1: vascular cell adhesion molecule 1
VEGF: vascular endothelial growth factor
